# Characterising donkey welfare challenges and opportunities associated with human activities and environmental factors in seven Kenyan counties

**DOI:** 10.1017/awf.2025.10057

**Published:** 2026-01-19

**Authors:** James Mutiiria Kithuka, Timothy Muthui Wachira, Wyckliff Ngetich, Joshua Orungo Onono

**Affiliations:** 1 Public Health Pharmacology and Toxicology, Faculty of Veterinary Medicine, University of Nairobi, Kenya; 2 Public Health Pharmacology and Toxicology, University of Nairobi College of Agriculture and Veterinary Sciences, Kenya; 3 Medicine and Surgery, Egerton University, Kenya

**Keywords:** Animal welfare, community education, *Equus asinus*, Kenya, livelihoods, SEBWAT

## Abstract

Donkeys (*Equus asinus*) play a vital role in supporting rural and peri-urban livelihoods across Kenya, yet their welfare remains poorly characterised and often compromised by human practices and environmental pressures. This study examined welfare challenges and opportunities across seven counties representing urban, high-potential, semi-arid, and arid production systems. A total of 392 donkeys were assessed using the Standardised Equine-Based Welfare Assessment Tool (SEBWAT), and structured interviews were conducted with owners to capture practices and environmental contexts. Data were analysed using descriptive statistics and multivariable logistic regression. Approximately 80% of donkeys exhibited at least one welfare concern. Common problems included poor body condition (48.2%), spinal pain (46.9%), lameness (33.4%), and mutilations (41.6%). Variation was observed across systems with donkeys in urban and high-potential areas showing more spinal sensitivity and behavioural distress. Key predictors of poor welfare included work type, terrain, limited veterinary access, housing, owner negligence, and donkey age ≥ 6 years. Owners prioritised community education (64.5%), veterinary outreach (52.0%), humane handling (27.3%), and improved access to feed and water (21.9%) as key interventions. These findings provide insights for designing targeted, context-specific interventions. A holistic approach addressing both human and environmental challenges is essential for safeguarding donkey welfare and protecting livelihoods.

## Introduction

Working donkeys (*Equus asinus*) are essential to the livelihoods of millions of people, particularly in low- and middle-income countries, where they provide critical draft power for transporting water, firewood, agricultural produce, and household goods (Grace *et al.* 2022; Raw *et al.* 2024). In Kenya, donkeys are especially important in rural and peri-urban settings, where they significantly reduce the burden of domestic and economic labour, particularly for women and children (Valette 2015; Gichure *et al.* 2020). Beyond transport, they are increasingly reared for production purposes, such as milk and meat (Hassan *et al.* 2022). Despite their vital contributions, donkeys remain economically undervalued, excluded from livestock development policies, and underserved by animal health systems (Geiger *et al.* 2020). This institutional neglect contributes to widespread welfare problems across the country.

Donkey welfare is shaped by a complex interplay of human behaviour, environmental conditions, and access to services (Carder *et al.* 2019; Haddy 2022). Common welfare issues include poor body condition, wounds, hoof deformities, lameness, ectoparasite infestations, and mutilations, all of which signal chronic neglect or harmful practices (Onono & Kithuka 2020; Abdifatah *et al.* 2023). Although donkeys are physiologically well-adapted to arid environments, they remain vulnerable to stressors such as heat, poor forage, long walking distances, and rugged terrain (Tassone *et al.* 2020; Ravichandran *et al.* 2023). In cold or wet climates, they are susceptible to illness and require appropriate shelter (Bukhari & Parkes 2023).

Kenya’s diverse production systems (urban, high-potential, semi-arid, and arid) are characterised by distinct ecological, social, and management conditions that shape donkey use and welfare outcomes. Environmental factors such as temperature extremes, terrain ruggedness, forage and water availability, and exposure to soft or hard surfaces greatly influence these outcomes. Donkeys in arid areas may walk long distances for water and market access, often in high temperatures and with limited feed, predisposing them to dehydration, fatigue, and hoof injuries. In contrast, those in urban settings face risks related to traffic congestion, overloading, concrete surface and inadequate shelter, which contribute to wounds, lameness, and stress. These inter-county variations influence the extent and type of welfare challenges encountered (Mellish & Stull 2025).

Another important determinant of donkey welfare is the type of work performed. In Kenya, donkeys are commonly used for cart pulling, pack transport and, to a lesser extent, ploughing. Each task brings with it unique physical demands. Transport of goods by cart is associated with harness wounds and joint strain; pack use can result in spinal injuries and poor balance; and ploughing may cause muscular fatigue due to prolonged exertion (Kithuka *et al.* 2025). Despite these risks, few studies in Kenya have systematically compared donkey welfare across counties or production systems.

Owner-related characteristics, including age, education, income, handling practices, and attitudes, further influence donkey welfare (Luna & Tadich 2019; White *et al.* 2023). Owners who lack resources or knowledge may perpetuate suffering, while those with positive attitudes invest in better care (Knight *et al.* 2004). Donkey-human interaction plays a key role in welfare; practices such as whipping, separation from bonded partners, and poor restraint methods cause pain and behavioural distress (De Santis *et al.* 2021; The Donkey Sanctuary 2023).

Although NGOs, such as Brooke, SPANA and The Donkey Sanctuary, have implemented community-based interventions, these often lack sufficient evidence linking human behaviour and environmental risk factors to observed animal welfare outcomes (Upjohn *et al.* 2014; Duckworth & Gross 2020). Behaviour change interventions are unlikely to succeed if they are not grounded in behavioural theory and informed by a contextual understanding of both owner motivations and environmental influences (Haddy 2022).

To address these knowledge gaps and provide the required information, this study examined the welfare of working donkeys in seven counties representing Kenya’s four main production systems. Welfare was assessed using six animal-based indicators adapted from the Standardised Equine-Based Welfare Assessment Tool (SEBWAT): body condition score; lesions; hoof shape; spinal sensitivity; ectoparasites; and evidence of mutilation. These indicators were selected because they provide a rapid yet comprehensive overview of the donkey’s physical health and are directly observable under field conditions, making them suitable for large-scale assessments. In addition, data were collected on owner demographics, donkey use, management practices, terrain, and access to veterinary services.

By triangulating animal-based, human, and environmental data, this research offers a context-specific analysis of donkey welfare and identifies actionable opportunities for improvement. The findings aim to inform policy, support targeted interventions, and strengthen the resilience of households that depend upon working donkeys across Kenya’s varied production systems.

## Materials and methods

### Study design and site selection

**Figure 1.**
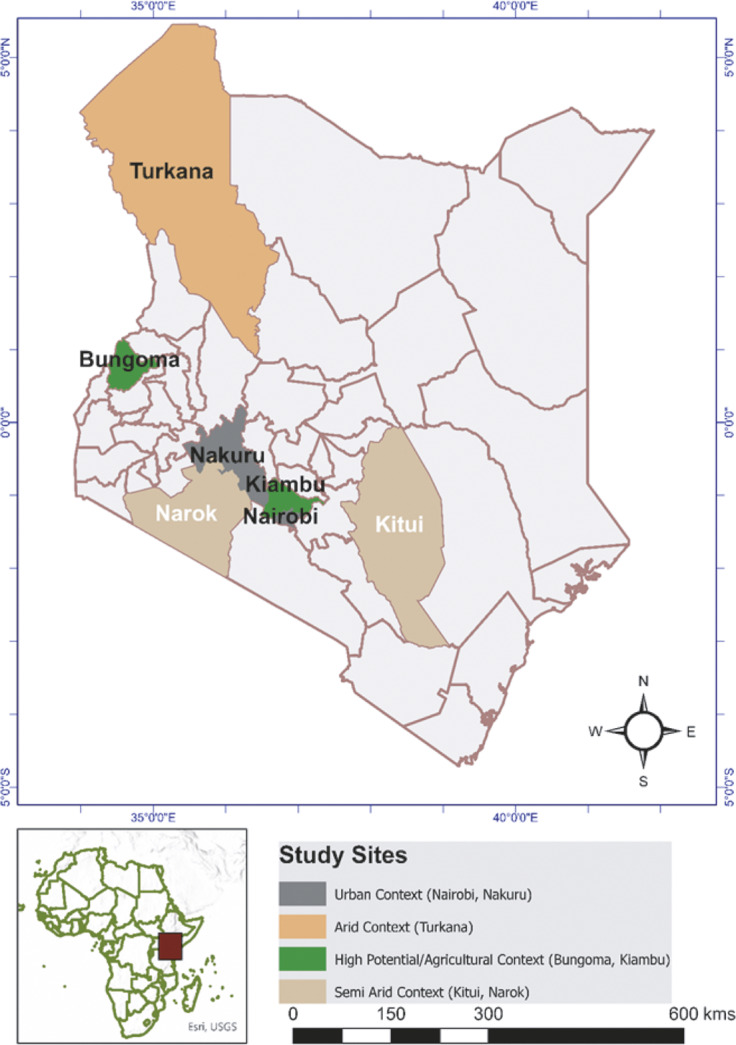
Map of study sites in Kenya selected for the donkey value chain study, categorised into urban, semi-arid, high potential, and arid production systems. Study sites were selected based on donkey density and management practices. The map was generated using ArcMap Version 10.8.2 (ESRI, CA, USA).

A cross-sectional study was conducted from November 1, 2024 to February 28, 2025 in seven Kenyan counties (Nairobi, Nakuru, Kiambu, Bungoma, Narok, Kitui, and Turkana) purposively selected to represent the country’s four major donkey production systems: urban; high-potential; semi-arid; and arid. The urban system is characterised by high human density, paved environments, and donkeys mainly used for transport of goods and water. The high-potential system includes agriculturally productive areas where donkeys support mixed farming and short-distance transport in poor steep soft soil terrain. The semi-arid system features seasonal rainfall and longer travel distances for water and markets, while the arid system is characterised by harsh climatic conditions and extensive mobility in search of grazing and water. Selection was based upon donkey population density, terrain type, predominant work patterns, and socio-ecological diversity. These counties provided representative variation in climate, infrastructure, and donkey management, allowing for meaningful comparison of welfare drivers across systems. Site locations are illustrated in Figure 1.

### Data collection approaches

#### Welfare assessment

Donkey welfare was assessed using SEBWAT, which comprises 40 animal-based indicators designed to evaluate the physical and behavioural welfare of working equids (Sommerville *et al.* 2018). These indicators capture key aspects of body condition, health, behaviour, and work-related lesions, providing a reliable and standardised framework for assessing donkey welfare under diverse field conditions. The assessors used a combination of visual inspection and tactile examination to assess eight core indicators: (i) body condition score; (ii) lesions (location, size, and severity); (iii) hoof shape and overgrowth; (iv) lameness and gait abnormality; (v) spinal sensitivity to palpation; (vi) ectoparasite infestation; (vii) mutilations such as ear notching or nose slitting; and (viii) behavioural responses including avoidance distance, reaction to handling, and general responsiveness to approach. These indicators were selected for their strong relevance to visible health and behavioural outcomes linked to owner practices and environmental stressors across different production systems.

Each assessment was carried out on a randomly selected donkey per owner using a mobile randomisation app (UXAPPS LTD 2023) to avoid selection bias. All three assessors, including the lead researcher (JMK), are certified SEBWAT assessors and qualified veterinarians. They also underwent a joint refresher training to ensure inter-observer reliability. Assessment procedures adhered strictly to SEBWAT and animal welfare assessment protocols (Farm Animal Welfare Advisory Council 2004).

### Questionnaire on human and environmental factor

Immediately following the welfare assessment, structured questionnaires were administered to respective donkey owners. Each questionnaire was matched to the welfare assessment of the same donkey, enabling correlation between owner/environmental data and animal welfare outcomes. The questionnaire, developed using the Capability, Opportunity, Motivation–Behaviour (COM-B) model of behaviour change (West & Michie 2020) was refined through pre-testing with 15 owners in Machakos and Nyandarua counties. It collected data on six domains: (a) owner characteristics (gender, age, education, income, ownership history); (b) donkey work type and intensity (e.g. cart, pack, plough; daily working hours; rest periods); (c) handling and care practices (use of whips, firing, equipment type, shelter access, veterinary care); (d) environmental stressors (terrain, drought, forage/water availability, seasonality); (e) community attitudes (perceived value of donkeys, local beliefs, support systems); and (f) gaps and opportunities for welfare improvement (training needs, veterinary access, Non-Governmental Organisation [NGO] presence).

Donkey owners were recruited in partnership with NGOs familiar with each study region: Caritas (Kitui), Life Skill Promoters (Kiambu and Nairobi), Ripple Effect (Bungoma), Farming Systems Kenya (Narok and Nakuru), and Agency for Pastoral and Development Kenya (Turkana).

### Data analysis

Welfare outcome was a binary variable: donkeys were classified as having ‘poor welfare’ if they exhibited scores ≥ 2 of the six animal-based SEBWAT indicators. Each welfare outcome was linked to the corresponding owner’s questionnaire responses, allowing for integrated analysis of donkey-level welfare status and associated human and environmental factors. Independent variables were coded as follows: Owner education (no formal, primary, secondary, tertiary); Veterinary access (yes/no); Work type (cart, pack, plough); Working hours (≤ 4, 5–8, > 8); Terrain (flat, hilly, mixed); Housing quality (adequate/inadequate); Income level (low, medium, high, based on self-reported tertiles); Perceptions (Likert responses dichotomised into agree/disagree).

Quantitative data were collected using Google® Forms, cleaned in Microsoft Excel®, and analysed in R version 4.1.2. Descriptive statistics (proportions, means) were generated using arsenal and tidyverse packages. Chi-squared tests were used for univariate analysis of welfare outcomes. Variables with a *P*-value ≤ 0.25 in the univariate analysis were retained for multivariate logistic regression using the glmer() function from the lme4 package in R. County was included as a random intercept to account for potential clustering of observations. Variance Inflation Factor (VIF) was used to assess multicollinearity; variables with VIF > 2.5 were excluded. The final model was selected based on Akaike Information Criterion (AIC). Statistical significance was set at *P* < 0.05. Intra-Class Correlation Coefficient (ICC) was calculated to assess variation attributable to county-level clustering.

### Ethical considerations

Ethical approval was obtained from the University of Nairobi Biosecurity, Animal Use and Ethics Committee (Ref: 2024/549) and the National Commission for Science, Technology, and Innovation (NACOSTI/P/24/36654). County-level permissions were secured from relevant veterinary departments. Informed verbal or written consent was obtained from all participants prior to data collection. All responses were anonymised and stored in accordance with the Data Protection rules in Kenya.

## Results

This section summarises results from 392 donkeys assessed across Kenya’s four production systems. Key findings include high overall poor welfare (80%) and significant differences across counties. Welfare indicators such as lesions, body condition, hoof shape, and mutilations varied by production system and were linked to both human and environmental factors. Multivariate analysis identified work type, housing, forage, terrain, and veterinary access as the strongest predictors.

### Overview of study population

This study assessed 392 donkeys and their owners from seven counties in Kenya, categorised into four production systems: urban (Nairobi, Nakuru); high potential (Kiambu, Bungoma); semi-arid (Kitui, Narok); and arid (Turkana). Owner demographics were broadly similar across systems, with a near-equal gender representation. Most respondents (41.1%) were aged between 36 and 49 years and the majority (68.6%) had attained primary education.

**Figure 2.**
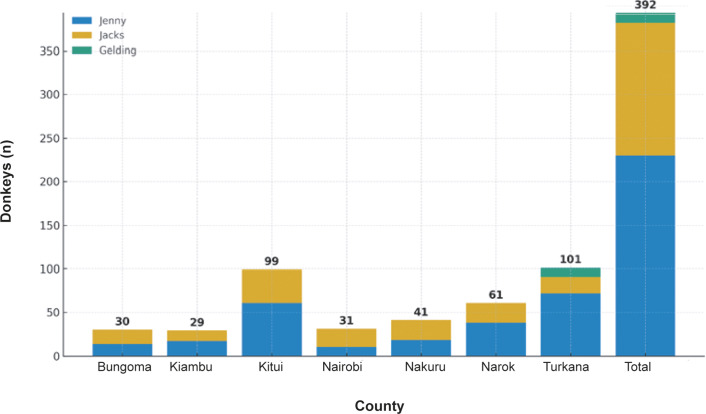
Distribution of donkey sex (male vs female) across seven counties in Kenya (Bungoma, Kiambu, Kitui, Nairobi, Nakuru, Narok, and Turkana), based on total sample size per county (n = 392 donkeys).

Regarding animal characteristics, the assessed donkeys varied in sex and distribution across counties and production systems. Of the total assessed donkeys, jennies were 58.7%, jacks 38.8%, and geldings 2.5%. Details regarding the total number of donkeys and their distribution by county are presented in Figure 2.

### Welfare outcomes and key indicators

**Figure 3.**
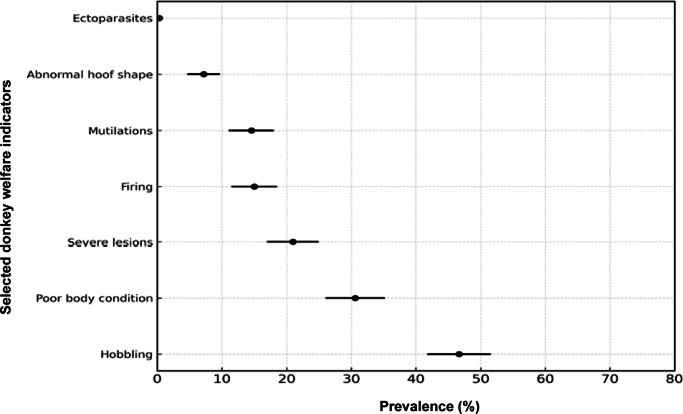
Forest plot displaying the prevalence and 95% confidence intervals of selected donkey welfare indicators (ectoparasites, abnormal hoof shape, mutilations, firing, i.e. use of hot iron to mark animals, severe lesions, poor body condition and hobbling) across seven Kenyan counties (Bungoma, Kiambu, Kitui, Nairobi, Nakuru, Narok, and Turkana), based on SEBWAT assessments of 392 donkeys conducted between November 1, 2024 and February 28, 2025.

A total of 392 donkeys were assessed across seven counties, of which 80% were classified as being in poor welfare condition and only 19.2% in good welfare. Welfare status was determined using seven animal-based indicators: poor body condition, severe skin lesions, abnormal hoof shape, ectoparasite infestation, mutilations, hobbling, and firing (use of hot instruments to mark or treat animals). The prevalence of these indicators varied considerably across counties (Table 1) and is further illustrated in Figure 3. A detailed breakdown of county-specific results is provided in Table S1 in the Supplementary material.

**Table 1.** County-level prevalence (%) of key welfare indicators among working donkeys (n = 392) assessed across seven counties in Kenya. The table presents the proportion of donkeys affected by each welfare concern within each county and the corresponding nation

Welfare indicator	Bungoma	Kiambu	Kitui	Nairobi	Nakuru	Narok	Turkana	National (%)
Poor body condition	63.3	24.1	67.6	9.7	17.1	25	8	30.6
Severe skin lesions	53.3	7	13.1	38.7	7	29.5	0	21.2
Abnormal hoof shape	40	0	6	4	2	0	0	7.4
Ectoparasite infestation	1	0	1	0	0	1	0	0.2
Mutilations	0	31	12.1	45.2	0	0	73.3	23
Firing	1	2	30	1	5	16	50	15
Hobbling	16.4	20.7	58.2	100	90.2	43	0	46.9

### Work type, load, and duration

The nature and intensity of donkey work varied widely across the seven counties and production systems. Overall, transport of goods by pack was the most common form of work, accounting for 69% of all donkeys assessed, while 31.0% were used for cart pulling. These differences were statistically significant (χ^2^ = 69.22; *P* < 0.001) and reflected the diversity of production environments. Pack work was dominant in the arid regions such as Turkana and Kitui, whereas cart pulling was more typical in urban and high-potential areas, including Nairobi, Kiambu, and Bungoma.

**Figure 4.**
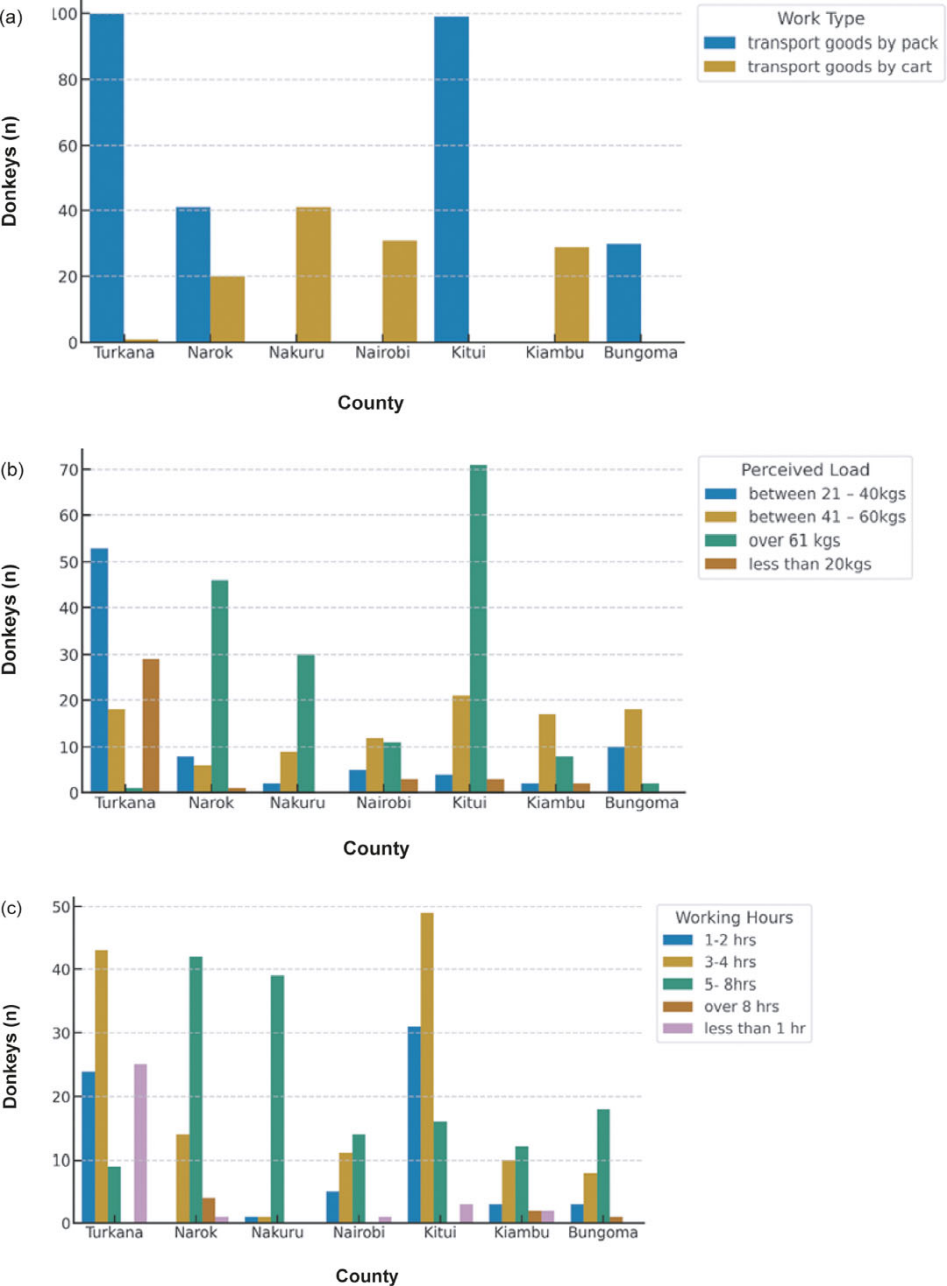
Distribution of donkey work characteristics showing (a) type of work performed (e.g. cart pulling, pack transport), (b) average load carried, and (c) daily working hours across seven counties in Kenya (Bungoma, Kiambu, Kitui, Nairobi, Nakuru, Narok, and Turkana), based on owner-reported data for 392 donkeys.

Variation was also evident regarding the perceived workload and the numbers of hours donkeys worked each day (*P* < 0.001). Heavier loads and longer working hours were more frequently reported in semi-arid and urban counties compared to high-potential areas. Figure 4 provides a summary of these patterns highlighting differences in work type, load, and daily working duration across counties while detailed county-level results are available in Table S2; see Supplementary material.

### Donkey management practices

Donkey management practices were evaluated across seven counties representing Kenya’s diverse production systems. Five key indicators were assessed: access to water; forage availability; donkey housing; grazing frequency; and access to veterinary services. Overall, the results revealed significant variation across regions.

**Figure 5.**
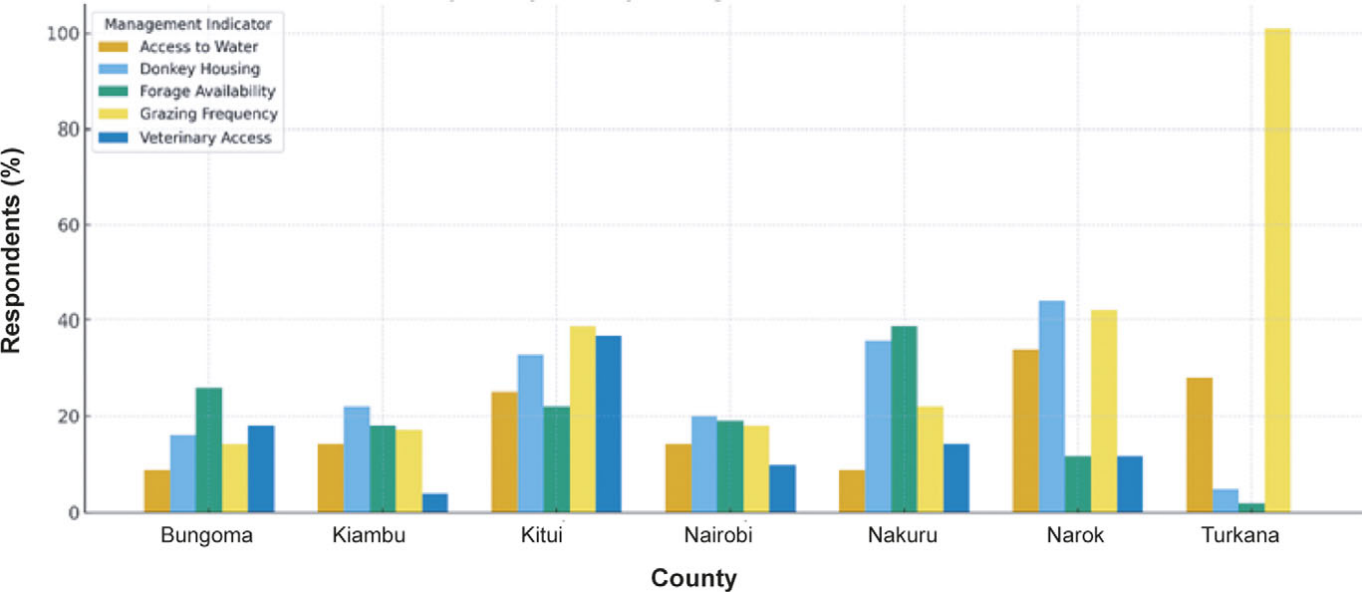
Comparative distribution of key donkey management practices — including feeding routines, housing conditions, and access to water — across seven counties in Kenya (Bungoma, Kiambu, Kitui, Nairobi, Nakuru, Narok, and Turkana), based on data from 392 donkey owners.

County-level differences in these management indicators are summarised in Figure 5, which highlights marked disparities in access to essential resources and husbandry practices. For instance, some counties showed relatively better access to water and veterinary services, while others experienced widespread shortages of these basic provisions. Variations were also evident in forage availability, housing conditions, and grazing frequency, reflecting the influence of environmental conditions and production systems.

### Mutilations, firing, and traditional practices

Traditional practices that negatively affect donkey welfare, including ear, muzzle, and tail mutilations, hobbling, and firing were observed across all seven counties, with notable regional variations. These practices ranged in frequency and severity, reflecting differing cultural norms and handling traditions across production systems.

**Figure 6.**
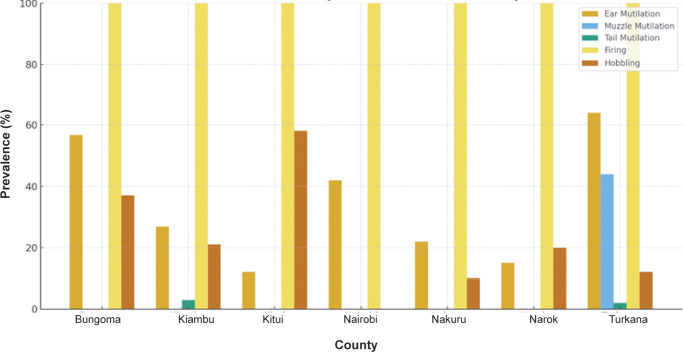
Prevalence of traditional donkey practices — including ear cutting, muzzle and tail mutilation, firing, and hobbling — across seven Kenyan counties (Bungoma, Kiambu, Kitui, Nairobi, Nakuru, Narok, and Turkana), based on observed and owner-reported data from 392 donkeys.

Figure 6 summarises the prevalence of these practices by county, illustrating marked differences in how donkeys are managed and treated. While some counties reported widespread use of certain practices, others showed relatively lower prevalence. The figure highlights the persistence of traditional methods that have important welfare implications.

### Behavioural and attitude assessments

A total of 392 donkeys were evaluated for behavioural and attitudinal responses during handling to complement the animal-based welfare indicators described earlier. The assessment focused on three key parameters adapted from SEBWAT: response to human approach; tolerance to chin contact; and general attitude.

**Figure 7.**
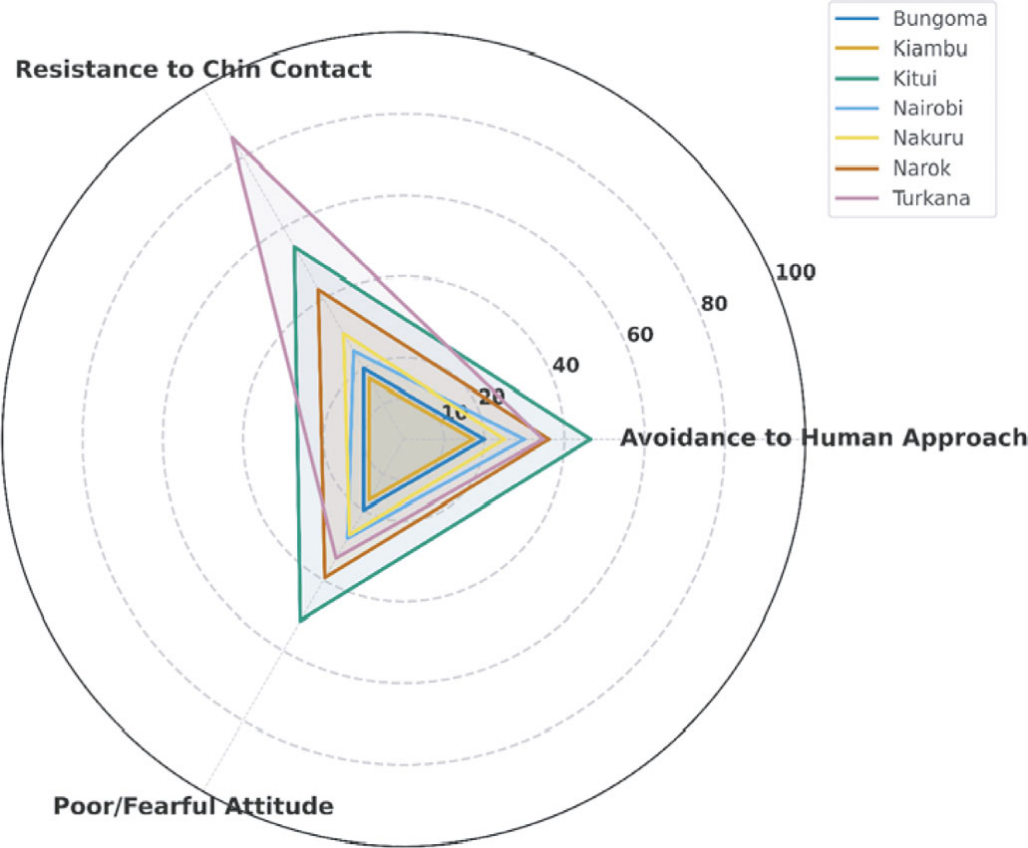
Radar plot showing the proportion of donkeys exhibiting avoidance to human approach, resistance to chin contact, and poor/fearful attitude across seven counties in Kenya. Higher values indicate greater expression of negative behavioural responses, suggesting reduced human-animal interaction quality or higher stress levels among donkeys.

The findings revealed clear variation across counties and production systems, reflecting differences in human-donkey interaction and handling practices. In certain counties, donkeys exhibited higher levels of avoidance and resistance, suggesting reduced familiarity, trust, or comfort during human contact. These patterns are illustrated in Figure 7, which presents a radar chart comparing behavioural responses across the seven counties. Counties with larger radial values indicate a higher proportion of donkeys showing negative behavioural traits such as avoidance, resistance, or fearful attitudes implying lower-quality human-animal interactions and potentially greater stress among working donkeys.

### Influence of human and environmental factors on donkey welfare

To better understand the drivers of poor welfare outcomes in working donkeys, we examined the associations between welfare status and a range of human and environmental factors using both univariate and multivariate logistic regression, complemented by Chi-squared tests. These analyses aimed to identify the most influential factors operating at both individual and structural levels.

A total of seven variables were initially subjected to univariate logistic regression (Table 2). Of these, six variables with a *P*-value ≤ 0.25 were selected for inclusion in the multivariate model: donkey housing; work type; access to veterinary services; terrain; forage availability; and owner age. Climate was excluded due to lack of statistical significance. The univariate analysis revealed that five factors were significantly associated with poor welfare, including donkey housing, work type, access to veterinary services, terrain, and forage availability. Owner age showed a borderline association.

**Table 2.** Results of univariate logistic regression analysis examining associations between selected human and environmental factors and poor donkey welfare status in seven Kenyan counties (Bungoma, Kiambu, Kitui, Nairobi, Nakuru, Narok, and Turkana). The analysis included 392 donkeys assessed using SEBWAT, with welfare status as the binary outcome. Reported are the odds ratios (OR), 95% confidence intervals (CI), and P-values for each predictor

Variable	Odds Ratio (OR)	95% CI Lower	95% CI Upper	*P*-value
Owner age	1.375	0.98	1.93	0.066
Donkey housing	1.376	1.15	1.646	< 0.001
Work type	3.399	2.027	5.699	< 0.001
Veterinary services access	1.343	1.097	1.645	0.004
Terrain	1.724	1.31	2.269	< 0.001
Access to forage	2.044	1.508	2.772	< 0.001
Climate	0.844	0.607	1.172	0.311

In the multivariate regression analysis (Table 3), model fit was evaluated using the Akaike Information Criterion (AIC), and multicollinearity was ruled out with Variance Inflation Factor (VIF) values below 2.5 for all retained variables. The final model included five variables, of which four emerged as statistically significant independent predictors of poor donkey welfare: inadequate donkey housing; limited access to veterinary services; rough or challenging terrain; and insufficient forage.

**Table 3.** Results of multivariate logistic regression analysis identifying key predictors of poor donkey welfare across seven counties in Kenya (Bungoma, Kiambu, Kitui, Nairobi, Nakuru, Narok, and Turkana). The analysis included 392 donkeys assessed using SEBWAT, with poor welfare status as the dependent variable. The table reports adjusted odds ratios (OR), 95% confidence intervals (CI), and P-values for each retained explanatory variable

Variable	Odds Ratio (OR)	95% CI Lower	95% CI Upper	*P*-value
Donkey housing	1.32	1.08	1.614	0.007
Veterinary services access	1.367	1.104	1.691	0.004
Terrain	1.423	1.023	1.978	0.036
Access to forage	1.609	1.15	2.251	0.005
Work type	1.799	0.912	3.55	0.09
Owner age	1.055	0.721	1.543	0.782
Climate	1.225	0.822	1.826	0.319

Although work type was strongly associated with poor welfare in the univariate analysis, it did not retain statistical significance in the final model. Similarly, owner age, despite being considered due to a borderline *P*-value, was not retained in the final multivariate model. The model’s Intra-Class Correlation Coefficient (ICC) was 0.13, suggesting that most variation in donkey welfare occurred at the individual animal level rather than being driven by county-level clustering.

Further analyses identified additional explanatory variables that influenced welfare. Donkey owners aged ≥ 50 years were significantly more likely to manage animals in good welfare condition compared to those under 18 years. Donkeys without a history of disease were also significantly more likely to exhibit good welfare status, and those kept in moderate housing structures were better off than those lacking housing altogether. Production system had a marked influence, with donkeys in arid regions substantially more likely to experience poor welfare compared to those in urban systems.

To complement the regression findings, Chi-squared tests were conducted to explore specific associations between human and environmental factors and individual welfare indicators (Table 4). Severe lesions were significantly associated with neglect, lack of water, and poor forage access. Mutilations were associated with owner gender, neglect, and a low perceived economic value of donkeys. Mutilation injuries also correlated with environmental stressors such as limited water access, forage scarcity, climatic hardship, and challenging terrain. Abnormal hoof shape was linked to terrain, water availability, and exposure to environmental changes like soft wet soils.

**Table 4.** Significant associations (*P* < 0.05) between donkey health and welfare parameters (severe lesions, mutilations, and hoof shape) and selected human and environmental factors across seven Kenyan counties. Results are based on Chi-squared (χ^2^) tests using data from 392 donkeys assessed through owner reports and welfare observations

Donkey health/welfare parameter	Human factors			Environmental factors		
	Factor	χ^2^	*P*-value	Factor	χ^2^	*P*-value
Severe lesions	Neglect	10.2	0.0013	Access to water	19.2	0.0007
				Poor forage	7.2	0.027
Mutilations	Owner gender	4.35	0.037	Access to water	13.3	0.010
	Neglect	22.35	< 0.001	Access to forage	45.44	< 0.001
	General perception	9.05	0.029	Climate impact	9.30	0.0096
	Donkey housing	12.65	0.013	Terrain effect	24.7	< 0.001
				Environmental change	13.95	0.00297
Hoof shape	General perception	8.24	0.41	Terrain effect	6.33	0.042
				Access to water	16.02	0.003
				Environmental change	7.96	0.047

### Identified opportunities for donkey welfare improvement

Questionnaire responses from donkey owners and stakeholders across the seven counties highlighted several opportunities for improving donkey welfare. The most frequently mentioned interventions included enhanced community education and awareness, improved access to veterinary services, promotion of humane handling practices, better nutrition, and regular health check-ups (Table 5).

**Table 5.** County-level distribution of respondent-identified opportunities to improve donkey welfare in Kenya, based on data from 392 donkey owners across seven counties (Bungoma, Kiambu, Kitui, Nairobi, Nakuru, Narok, and Turkana). The table shows the proportion of owners supporting specific interventions — such as education, humane handling, improved housing, and access to veterinary services—with Chi-squared (χ^2^) tests used to assess statistical differences between counties. Only opportunities with significant variation (*P* < 0.05) are included

Existing opportunities	Bungoma (n = 30)	Kiambu (n = 29)	Kitui (n = 99)	Nairobi (n = 31)	Nakuru (n = 41)	Narok (n = 61)	Turkana (n = 101)	Total (n = 392)	*P*-value
Community education and awareness									< 0.001
Yes	46.7%	41.4%	51.5%	67.7%	73.2%	83.6%	73.3%	64.5%	
No	53.3%	58.6%	48.5%	32.3%	26.8%	16.4%	26.7%	35.5%	
Humane handling of donkeys									< 0.001
Yes	20.0%	24.1%	6.5%	25.8%	14.6%	44.3%	44.6%	37.0%	
No	80.0%	75.9%	53.5%	74.2%	85.4%	55.7%	55.4%	63.0%	
Strengthening laws and regulations									0.009
Yes	16.7%	13.8%	24.2%	12.9%	43.9%	21.3%	33.3%	23.7%	
No	83.3%	86.2%	75.8%	87.1%	56.1%	78.7%	66.3%	76.2%	
Regular health checkup									0.003
Yes	16.7%	13.8%	30.3%	25.8%	26.8%	45.9%	43.6%	33.2%	
No	83.3%	86.2%	69.7%	74.2%	73.2%	54.1%	56.4%	66.8%	
Improve housing conditions									< 0.001
Yes	16.7%	20.7%	12.1%	16.1%	46.3%	16.4%	11.9%	20.0%	
No	83.3%	79.3%	87.9%	83.9%	53.7%	83.6%	88.1%	82.4%	
Increase access to veterinary services									0.002
Yes	66.7%	58.6%	75.8%	48.4%	53.7%	47.5%	70.3%	63.5%	
No	33.3%	41.4%	24.2%	51.6%	46.3%	52.5%	29.7%	36.5%	
Providing better nutrition									< 0.001
Yes	53.3%	34.5%	69.7%	22.6%	9.8%	16.4%	27.7%	36.7%	
No	46.7%	65.5%	30.3%	77.4%	90.2%	83.6%	72.3%	63.3%	

Community education and awareness emerged as the top priority, most frequently cited intervention across all production systems. Owners, particularly from Narok, Nakuru, and Turkana, expressed a strong need for training on proper husbandry, feeding, handling techniques, and prevention of injuries. Even in counties like Nairobi and Kitui, respondents noted that awareness campaigns could significantly improve welfare outcomes.

Access to veterinary services was emphasised by nearly two-thirds of all respondents, with particularly strong calls from Kitui and Turkana. Donkey owners reported difficulties accessing timely treatment for wounds, infectious diseases, and parasitic infestations. Suggested measures included outreach programmes, subsidised medicines, and more frequent mobile veterinary clinics.

Humane handling was another key area highlighted in Narok and Turkana, where welfare assessments revealed high incidences of tethering injuries, hobbling, and nose mutilations. In Nairobi and Kiambu, concerns focused more on overloading and harsh training methods. Respondents proposed community dialogues, peer education, and stricter enforcement of animal welfare regulations to address these issues.

Regular health check-ups were supported by one-third of respondents overall, especially in Narok and Turkana. Owners emphasised the importance of periodic deworming, physical examinations, and monitoring of common conditions such as lameness and ectoparasites. This aligns with earlier observations linking poor welfare to untreated injuries and chronic health conditions.

Improved nutrition, especially access to quality feed and forage was another frequently mentioned opportunity. Counties such as Kitui, Bungoma, and Turkana reported feed scarcity as a key driver of poor body condition. Participants suggested establishing communal fodder banks, pellet feed production, rotational grazing areas, and improved storage methods to mitigate seasonal shortages.

Other areas, such as strengthening of animal welfare laws (23.7%) and improving housing structures (20%), were also noted as important but received comparatively less emphasis. Counties like Nakuru, Turkana and Kitui stood out for their support of legal reform, possibly reflecting challenges related to donkey exploitation and illegal slaughter. Improving shelter and protection from extreme weather was cited by several respondents, particularly in Nakuru. Only one donkey owner from Bungoma mentioned anti-stock theft security personnel. The reporting of these welfare opportunities was statistically significant across the seven counties, as summarised in Table 5.

## Discussion

This study presents a comprehensive overview of donkey welfare across Kenya’s four major production systems, highlighting how animal suffering is shaped by a combination of human decisions, environmental pressures, and access to services (Pritchard *et al.* 2018; Gichure *et al.* 2020; Yalew *et al.* 2024; Mellish & Stull 2025). African field studies show that harsh environmental conditions (feed/water scarcity, extreme heat) and workload/terrain significantly worsen body condition and lameness, while traditional harnessing and owner handling practices increase wound prevalence (Eriso *et al.* 2023). By applying a mixed-methods approach across diverse counties, the findings not only reveal regional disparities but also unpack the underlying social and structural drivers of poor welfare. Importantly, the study moves beyond simple documentation of injuries or poor body condition and begins to map how specific risk factors, such as work type, terrain, veterinary access, and owner behaviour, converge to determine welfare outcomes.

Counties such as Turkana and Kitui stood out as critical hotspots for poor welfare. In Turkana, the prevalence of donkey mutilations (73.3%), reports of muzzle injuries (44.6%), and widespread use of firing practices reflected both the harsh environmental conditions and deeply rooted ethno-veterinary traditions. Kitui had the highest proportion of donkeys with poor body condition (67.7%) and a notably high rate of hobbling (58.6%). These findings reflect a broader pattern observed in drought-prone, pastoralist regions where donkeys are indispensable for accessing water and fuel, yet are frequently exposed to harmful practices due to lack of veterinary support and prevailing cultural norms (Valette 2015). Comparable patterns have been documented in Ethiopia’s Afar and Oromia regions, where nostril slitting, hobbling, and firing persist as culturally endorsed interventions for managing working donkeys (Derbib *et al.* 2024). Similar welfare challenges have also been reported in other African countries, including Tanzania, Mali and Kenya, where resource scarcity, cultural practices, and limited veterinary access contribute to persistent injuries and poor body condition (McLean *et al.* 2012; Rayner *et al.* 2018; Gichure *et al.* 2020).

Counties such as Nairobi, Nakuru, and Narok have shown relatively better donkey welfare in our study, higher body condition scores, fewer lesions and more positive behavioural responses (approachability and tolerance to chin contact). Nakuru’s, relatively high housing adequacy (46.3%) coupled with Narok’s stronger uptake of regular health checks (45.9%) indicate that basic infrastructure and service access support better welfare outcomes. These Kenyan patterns are consistent with evidence from elsewhere in Africa; region-wide and country studies link improved housing, community education and veterinary outreach to reduced wounds and better body condition (McLean *et al.* 2012), while surveys in Ethiopia and Tanzania document high prevalences of lesions and poor body condition where services and owner support are limited (McLean *et al.* 2012; Yalew *et al.* 2024). In South Africa, stronger local enforcement and coordinated welfare programmes in some provinces have been associated with reduced abuse and improved service access compared with less-regulated settings such as parts of Latin America or South Asia (Curran & Smith 2005; Province of Eastern Cape 2022; Ortega *et al.* 2024). Together, these studies indicate that structural factors; housing, veterinary services, enforcement, and targeted owner interventions are key drivers of spatial differences in donkey welfare across Africa, not simply geography or workload alone.

The influence of work type emerged as particularly significant in shaping welfare risks. While cart-pulling was the most common form of labour nationally (48.5%), it was strongly associated with poor welfare in univariate regression (OR = 3.40; *P* < 0.001). Donkeys pulling carts are often exposed to heavy loads, poor harnessing, traffic stress, and prolonged work hours, especially in urban and high-potential counties like Nairobi, Nakuru, and Kiambu. Although this association weakened in the multivariate model (*P* = 0.090), the physical demands of cart work remain a major welfare concern. Similar findings have been reported across Africa, where donkeys used for cart transport experience frequent wounds, lameness, and poor body condition due to overloading, ill-fitting harnesses, limited rest, and lack of veterinary support (Geiger *et al.* 2020). Comparable risks associated with overloading and prolonged working hours have also been observed in Ethiopia and Nigeria, where inadequate harnessing and lack of rest are leading causes of lesions and lameness (Mshelia *et al.* 2023; Derbib *et al.* 2024).These results parallel global observations from India’s brick kilns, where comparable patterns of overwork and soft tissue injuries have been documented (Kubasiewicz *et al*. 2022).

More broadly, the final multivariate model confirmed that four factors were independently associated with poor welfare: inadequate housing (OR = 1.32; *P* = 0.007); lack of veterinary access (OR = 1.37; *P* = 0.004); rough terrain (OR = 1.42; *P* = 0.036); and insufficient forage (OR = 1.61; *P* = 0.005). Each of these reflects systemic deficiencies in the donkey production environment. Terrain-related stress was especially evident in Kitui, Narok, and Turkana — counties where hoof abnormalities and spinal sensitivity were more common, likely due to the long distances travelled by donkeys over uneven paths. These findings resonate with evidence from mountainous regions of Morocco and Afghanistan, where steep and rocky terrain is a known cause of lameness and hoof overgrowth in working equids (Sells *et al.* 2010; Bukhari *et al.* 2022).

Interestingly, owner age was also a predictor of welfare: donkeys owned by individuals aged ≥ 50 years were significantly more likely to be in good condition compared to those managed by younger handlers (OR = 0.05; *P* = 0.047). This association may reflect greater empathy, accumulated experience, or lower economic pressure among older owners. Similar generational patterns have been observed in Niger and Senegal, where elderly herders prioritise animal well-being more consistently than their younger counterparts, who often face economic strain and limited awareness of welfare best practices (McLean *et al.* 2012). Beyond age, gender roles also appear to influence welfare outcomes. In many Kenyan communities, men primarily use donkeys for heavy transport and commercial purposes, while women tend to use them for domestic tasks such as fetching water and firewood. Studies indicate that donkeys managed by women may experience lighter workloads and more consistent care, reflecting a closer emotional bond, whereas those used by men are often subjected to greater work intensity and risk of injury (Valette 2015; Haddy *et al.* 2021). These findings underscore the need to consider both age and gender dimensions when designing welfare interventions for working donkeys.

Another strong signal from the analysis was the role of owner neglect characterised by failure to provide adequate care, feeding, or timely treatment in causing severe lesions and mutilations. Chi-squared tests revealed strong associations between neglect and lesions (χ^2^ = 10.2; *P* = 0.001), as well as with mutilations (χ^2^ = 22.4; *P* < 0.001). These findings are deeply concerning given the emotional and physical trauma such practices entail. These injuries were frequently co-located with poor environmental conditions — especially forage and water scarcity, which were predominant in Turkana and Kitui. As documented in Egypt, failure to provide shelter during extreme heat also contributes to dehydration, exhaustion, and early mortality in working equids (Farhat *et al.* 2020).

The study found that donkey owners were able to identify practical and relevant opportunities for welfare improvement. The most commonly proposed interventions were community education (64.5%), improved access to veterinary services (63.5%), and promotion of humane handling practices (37.0%). These priorities are directly aligned with the statistical predictors of poor welfare identified in the study, suggesting that owners are aware of the causes of suffering and open to change. However, less attention was given to shelter improvement (17.6%) and legal enforcement (26.0%), despite their known importance in the literature (Haddy *et al.* 2020).

The variation in perceptions between counties was also notable. Turkana and Kitui, despite experiencing the most severe welfare outcomes, were among the strongest advocates for improved veterinary access and regular health check-ups, reflecting a clear demand for support. Meanwhile, owners in Nakuru and Nairobi placed greater emphasis on legal reforms, possibly in response to the threats posed by donkey theft and illegal slaughter in urban settings.

This study reinforces the idea that donkey welfare is not solely a function of poverty or neglect, but defined by a complex interplay of environmental pressures, owner knowledge, structural access to services, and cultural norms. The persistence of welfare challenges despite owners’ care and concern indicates that awareness alone is insufficient without supportive systems and resources. Limited veterinary access, weak policy enforcement, and inadequate welfare integration into local governance continue to undermine progress. Across Africa, weak enforcement of welfare legislation and limited veterinary infrastructure have been identified as key systemic constraints (Gichure & Olayide 2024; Kithuka *et al.* 2025). Strengthening these structures through policy integration remains central to sustainable welfare improvement. Addressing these issues requires a holistic, systems-based approach that combines behaviour change, institutional strengthening, and community empowerment to sustainably improve both animal welfare and human livelihoods (Pritchard *et al.* 2018).

### Animal welfare implications

This study reveals that 80% of donkeys across surveyed counties in Kenya suffer from poor welfare, primarily as a result of human-related factors such as poor housing, overwork, mutilations, and inadequate veterinary care, compounded by environmental stressors like rough terrain and limited forage. The findings underscore how human practices, especially those driven by socioeconomic constraints, directly influence the health, behaviour, and overall well-being of donkeys (Kubasiewicz *et al*. 2023). To improve their welfare, it is important to educate owners, provide better veterinary services, promote kind handling, and improve the donkeys’ living and working environments.

## Conclusion

This study confirms that donkey welfare in Kenya is significantly shaped by work type, terrain, owner characteristics, and access to services, with wide disparities across production systems. Poor welfare outcomes were most evident in arid and semi-arid areas such as Turkana and Kitui, where harmful practices — including firing, hobbling, and mutilations — were prevalent, and access to essential resources like forage, water, housing, and veterinary care was limited. In contrast, better management indicators were more frequently reported in urban and high-potential counties such as Nairobi, Kiambu, and Nakuru, though welfare challenges remained.

Key predictors of poor welfare included inadequate housing, limited veterinary access, rough terrain, and forage scarcity. Encouragingly, donkey owners recognised the need for education, humane handling, and improved veterinary support, though structural issues like housing and policy enforcement received less attention.

Addressing these challenges requires a holistic approach that combines community-led behaviour change, strengthened service delivery, and supportive policy frameworks to enhance donkey welfare and safeguard the livelihoods that depend upon them.

## Supporting information

10.1017/awf.2025.10057.sm001 Kithuka et al. supplementary material Kithuka et al. supplementary material

## Supplementary material

The supplementary material for this article can be found at http://doi.org/10.1017/awf.2025.10057.
